# Ethnicity and detention: are Black and minority ethnic (BME) groups disproportionately detained under the Mental Health Act 2007?

**DOI:** 10.1007/s00127-016-1181-z

**Published:** 2016-02-17

**Authors:** Ruchika Gajwani, Helen Parsons, Max Birchwood, Swaran P. Singh

**Affiliations:** Institute of Health and Wellbeing, Yorkhill Hospital, University of Glasgow, Caledonia House, Glasgow, G3 8SJ UK; Cancer Research, Warwick Medical School, Coventry, CV4 7AL UK; Mental Health and Wellbeing, Warwick Medical School, Coventry, CV4 7AL UK

**Keywords:** Ethnicity, Mental Health Act, Detention, BME, Transcultural psychiatry

## Abstract

**Purpose:**

There is substantial evidence to suggest that Black and minority ethnic (BME) patients are disproportionately detained under the Mental Health Act (MHA). We examined ethnic differences in patients assessed for detention and explored the effect of ethnicity after controlling for confounders.

**Methods:**

A prospective study of all MHA assessments conducted in 1 year (April 2009–March 2010) within Birmingham and Solihull Mental Health Foundation Trust, UK. Proportion of assessments and detentions within denominator population of service users and regional populations were calculated. Multiple regression analysis was conducted to determine which variables were associated with the outcome of MHA assessment and the role of ethnicity.

**Results:**

Of the 1115 assessments, 709 led to detentions (63.58 %). BME ethnic groups were statistically more likely to be assessed and detained under the MHA as compared to Whites, both in the service user and the ethnic population estimates in Birmingham, UK. MHA detention was predicted by having a serious mental illness, the presence of risk, older age and living alone. Ethnicity was not associated with detention under the MHA with age, diagnosis, risk and level of social support accounted for.

**Conclusion:**

The BME ‘disproportionality’ in detention rates seems to be due to higher rates of mental illness, greater risk and poorer levels of social support rather than ethnicity per se.

## Introduction

Compulsory psychiatric admission has been associated with a diagnosis of schizophrenia and related disorders, risk, gender, unemployment, ethnicity, lack of social support, dangerousness and differences in legal criteria for involuntary admissions across countries [1–5]. In England, involuntary psychiatric admissions *per annum* increased by 20 % from 1996 to 2006, with over 50 % of inpatients being treated for psychosis and substance misuse disorders [6]. More specifically, Black and minority ethnic (BME) patients have consistently been reported to be disproportionately detained under the Mental Health Act, 1983 (MHA) [7, 8]. Detentions amongst BME groups is statistically greater than those from a White British ethnicity amongst adolescent psychiatric admissions [9], first-episode psychosis [10] and severe and enduring mental health conditions [4], in civil [8, 11] and forensic psychiatric services [12, 13]. Some studies have found that ethnic excess in compulsory admission reduces or is eliminated once confounding factors such as age, gender, diagnosis, risk and pathways to care are controlled for [4, 8, 14, 15]. However, in other studies BME status remained an independent predictor of psychiatric detention [2, 16], with ethnic variations between BME groups in experiences of mental health services [17]. Recent work investigating factors that predict MHA assessments and detentions in the UK is revealing a complex and multi-faceted relationship between ethnicity and detention. Amongst women experiencing mental health crisis [14] and first-episode psychosis [18] in London, high rates of compulsory detention in BME women were partially explained by poor help-seeking behaviour and differences in pathways to care. In a longitudinal study of all adolescent psychiatric admissions in London from 2001 to 2010, Corrigall and Bhugra [15] found that adolescents from a Black ethnic group with a diagnosis of psychosis were three times more likely than the White British group to be detained, but there was no ethnic variation in non-psychotic detentions with statistical significance.

To understand where the BME ‘disproportionality’ occurs, we explored the higher risk of detention using different denominator populations in Birmingham, UK: the population assessed under the MHA within the base population and the service user population. We wanted to determine whether all BME poeople and service users are at a higher risk of detention, or only the subgroup that meets the specific criteria for being detained—having a serious mental illness, requiring treatment, being at risk, and there being no alternative to treatment under MHA. Most studies of MHA use in BME populations are on detained cohorts, but this does not allow exploration of variables related to detention which can only be explored by evaluating the outcomes of all MHA assessments [8] and comparing those detained with the rest. To the best of our knowledge, the Department of Health-funded AMEND [4] and ENRICH studies led by the R&D unit in Birmingham were the first to investigate data on who gets assessed under the MHA and factors involved in the outcome of those assessments.

### Aims of the study

The aims of this study were twofold. To examine ethnic differences in the proportion of individuals undergoing MHA (2007) assessments and detentions in a given a year, within two denominator populations; mental health service users in Birmingham and the regional BME population. Secondly, to assess clinical and socio-demographic factors associated with the outcome (detention vs. non-detention) of all MHA assessments during the study period.

## Materials and methods

### Procedure

This research was part of the Department of Health-funded ENRICH (Ethnicity, Detention and Early Intervention: Reducing Inequalities and Improving outcomes for BME patients) study conducted over a period of 4 years (http://www.journalslibrary.nihr.ac.uk/news/ethnicity,-detention-and-early-intervention-reducing-inequalities-and-improving-outcomes-for-black-and-minority-ethnic-patients-the-enrich-programme,-a-mixed-methods-study-publishes-in-programme-grants-for-applied-research). Data were obtained from MHA (2007) assessments between April 2009 and March 2010, including demographic characteristics, previous MHA assessments, risk factors, substance misuse, diagnosis, outcome of assessments including community alternatives. Ethics approval was granted by Warwickshire Research Ethics Committee (WREC), Research and Development Department (R&D) within the mental health trust and Birmingham City Council (BCC). In accordance with the MHA (2007), details of all assessments, irrespective of the outcome were recorded by Approved Mental Health Professionals (AMHPs) on a two-part legal documentation (i.e. SS101 and CR6B), which included a structured monitoring form (Part I) and a detailed assessment record (Part II). Part II of the assessment records information on details of last/previous admission, circumstances leading to assessment/reassessment, record of interviews and discussions with the service user, and nearest relatives if present, as well as with doctors and other professional staff, assessment of risk, social situation and reason for decision including consideration of alternatives to compulsory admission.

Prior to data collection, the study was presented to over a hundred AMHP’s in the region, with periodic contact to request adequate data recording. The lead researcher (RG) made weekly contact with clinical teams, including on-call clinicians, crisis resolution/home treatment teams, forensic services, inpatient wards, emergency duty teams, and community teams to identify all MHA assessments conducted in the previous week. To ensure consistent and reliable data collection, a consistent coding regime was used and all assessments were cross-checked with patient electronic database to ensure minimum missing data.

### Instrument

Data were collected under the following headings: (1) *patient characteristics* sociodemographic variables such as age, gender, ethnicity, residential status, level of community and social support and clinical variables including diagnosis, presence of risk, substance misuse, risk factors, (2) *setting of the assessment* where the assessment was conducted (venue, day, time), whether a carer/family member was present and notified, (3) *service characteristics* local bed availability, availability of alternatives to detention, provision of specialist outreach service, (4) *assessment outcome* detained or not detained, specification of detention, alternative community treatment being available (example, ‘home treatment team’ managing risk in the community), voluntary admission from the service user and community treatment orders.

### Definitions

#### Ethnicity

Ethnicity was recorded through the MHA assessment and counter checked for any errors through electronic records if available. Six broad ethnic groups were created for the purpose of secondary analysis: White (White British/Irish/White-Other), Asian Pakistani (Asian/Asian British Pakistani), African Carribean (African/British African Carribean), Black African (Black/Black British African/African-Other), Asian Indian (Asian/Asian British Indian), Asian Bangladeshi (Asian/Asian British Bangladeshi). Individuals who had refused to give a self-assigned ethnicity and for whom no ethnicity was recorded were classed as missing and removed from the analysis.

#### Mental Health Act assessment

MHA assessment was defined as a clinical encounter where an AMHP had been involved or invited, or where at least one medical recommendation has been completed, regardless of the outcome of the assessment (detention, voluntary admission or no admission).

#### Risk

Data on risk were obtained from MHA monitoring forms in the following categories: self-harm, self neglect, deterioration in mental state, harm to other people, harm to property, and harm to vulnerable others. Where data on risk were not recorded, no risk was assumed for that category, unless all six risks were missing. Where all risk data were missing, the case was excluded from analysis.

#### Diagnosis

Psychiatric diagnosis (ICD-10) were categorised under four disorders: *psychopathic disorder* F10–19: mental and behavioural disorders due to psychoactive substance use; F60–69: disorders of adult personality and behaviour; *Mental impairment* F70–79: mental retardation; F80–89: disorders of psychological development; *Mental illness* F00–09: organic, including unspecified organic or symptomatic mental disorders; F20–29: schizophrenia, schizotypal and delusional disorders; F30–39: mood (affective) disorders F40–49: neurotic, stress-related and somatoform disorders F50–59: behavioural syndromes associated with physiological disturbances and physical factors; *Multiple psychiatric diagnoses* (more than one ICD 10 code). Psychiatric diagnoses for all assessments were confirmed from medical records.

### Data analysis

All data were encoded and analysed using statistical analysis software (SPSSv21). First, overall assessments and their outcome were coded through a ‘unique individual ID’ to calculate descriptive statistics. This variable was used to establish the number of assessments and detentions for each person in the proposed year of study. For the population calculations, the number of assessments was calculated as the number of unique people in the database; if a person was assessed multiple times, they were coded as ‘one assessment’. For the population calculations, the outcome of the assessment was calculated with the unique individual in the database: (1) if a person was assessed multiple times and detained at any one of them, it was coded as ‘detention’, (2) if a person was assessed multiple times, and detained each time, it was coded as ‘detention’, (3) if a person was assessed multiple times, and never detained, it was coded as ‘no detention’.

Second, Chi square analysis was conducted to investigate the differences in proportion of assessment and detentions between the six largest ethnic groups with the White British/White Other ethnic group within two denominator population; service users in Birmingham and the ethnic population estimates of Birmingham in 2009 (http://www.birmingham.gov.uk/). To allow for Chi squared tests to be carried out for the small number of Asian Bangladeshi group, Fisher’s exact test was used [19]. For tables larger than two by two categories, *p* values were simulated using a Monte Carlo Simulation with a thousand replicates. Adjustment for multiple testing was applied using Bonferroni correction (i.e. adjustment for 15 comparison tests).

Thirdly, univariate analyses were conducted to identify socio-demographic and clinical variables that statistically differed between ethnic groups. Ethnicity data were pooled into broad Black, White and Asian groups due to the small numbers in some BME groups. Six variables thus identified were checked for co-linearity using Pearson’s correlation with each of the five other factors, then used to model detention. A logistic regression model was constructed to investigate the relationship of the independent variables with the outcome of a patient’s mental health assessment (either ‘resulted in detention’ or ‘no detention’). Variables were entered into the model and identified as categorical where appropriate. Models were constructed for each variable singly (single regression), and together (multiple regression). For the multiple regression model, the ENTER method was used to force inclusion of all factors into the final model where model coefficients could be easily compared. For all models, variables with more than two categories were tested for significance as combined factors using an omnibus test, which allows the overall effect of the variable to be captured alongside the effects of each category. Odds ratios and 95 % confidence intervals were computed for each individual category.

## Results

### Descriptive

Between April 2009 and March 2010, 1115 MHA assessments were conducted in Birmingham on 863 individuals (some of whom were assessed more than once), with a mean age of 40.12 (SD = 14.75) and 60.3 % men. Of the 1115 assessments, 709 led to detentions (63.58 %). Of the 861 assessments from April to December 2009, 559 (65 %) led to detentions, and 151 (59.4 %) of the 254 assessments led to detentions from January to March 2010. Of the individuals assessed (*n* = 863), 443 (51.3 %) had previous hospital admissions and substance misuse was recorded for 295 (34.2 %) cases. The largest proportion of diagnostic composition of those assessed was schizophrenia, schizotypal and delusional disorders (F20–29) (48.1 %), mood and affective disorder (F30–F39) (25.3 %) and disorders of adult personality and behaviour (F60–F69) (4.8 %).

Six cases were removed from secondary analysis due to missing ethnic data. The ethnic profile of individuals assessed (*n* = 857) was White (51.1 %), Asian Pakistani (14.9 %), African Carribean (14 %), Black African (7 %), Asian Indian (5.6 %), Asian Bangladeshi (1.6 %), Mixed ethnicity (2.6 %), Other (3.2 %). Of the 857 individuals assessed, 591 were detained; a proportion assessed and detained more than once in a year.

### Ethnicity and sample characteristics

Table 1 describes the key study characteristics of the six largest ethnic groups at the time of assessment. Of the individuals assessed, gender, employment risk and assessment outcome were not found to be associated with ethnicity with statistical significance.

**Table 1:** Socio-demographic and clinical profile of assessments by ethnicity, with simulated *p* value (Monte Carlo simulation with 1000 replicates)

	White British/other (*n* = 438)	Asian Pakistani (*n* = 126)	Asian Indian (*n* = 47)	Asian Bangladeshi (*n* = 16)	African Caribbean (*n* = 120)	Black African (*n* = 62)	Fisher’s exact test
Gender [*n* (%)]							
Male	250 (57.1)	89 (70.6)	26 (55.3)	12 (75)	70 (58.3)	34 (54.8)	*p* = 0.07
Female	188 (42.9)	37 (29.4)	21 (44.7)	4 (25)	50 (41.7)	28 (45.2)	
Age [*n* (%)]							
Under 35	146 (33.3)	76 (60.8)	16 (34)	14 (87.5)	48 (40)	35 (56.5)	*p* < 0.001
Over 35	292 (66.7)	49 (39.2)	31 (66)	2 (12.5)	72 (60)	27 (43.5)	
Living status [*n* (%)]							
Alone	181 (50.4)	16 (15.5)	12 (26.7)	2 (16.7)	63 (62.4)	24 (48)	*p* < 0.001
With others	164 (45.7)	81 (78.7)	29 (64.4)	10 (83.3)	37 (36.6)	22 (44)	
NFA/homeless	14 (3.9)	6 (5.8)	4 (8.9)	0 (0)	1 (1)	4 (8)	
Employment [*n* (%)]							
Unemployed	267 (75.2)	89 (86.4)	34 (77.3)	10 (83.3)	85 (86.7)	41 (85.4)	*p* = 0.08
Other	88 (24.8)	14 (13.6)	10 (22.7)	2 (16.7)	13 (13.3)	7 (14.6)	
Legal status [*n* (%)]							
Community/none	221 (50.5)	62 (49.2)	29 (61.7)	6 (37.5)	56 (46.7)	26 (42)	*p* < 0.01
Hospital informal	43 (9.8)	6 (4.8)	3 (6.4)	1 (6.3)	6 (5)	2 (3.2)	
Section 2	54 (12.3)	21 (16.7)	5 (10.6)	7 (43.8)	18 (15)	10 (16.1)	
Section 3	28 (6.4)	10 (7.9)	3 (6.4)	0 (0)	9 (7.5)	6 (9.7)	
Section 135/136	36 (8.2)	6 (4.8)	4 (8.5)	0 (0)	13 (10.8)	10 (16.1)	
CTO/CTO recall	7 (1.6)	10 (7.9)	1 (2.1)	1 (6.3)	11 (9.2)	3 (4.8)	
Other	49 (11.2)	11 (8.8)	2 (4.3)	1 (6.3)	7 (5.8)	5 (8.1)	
Outcome [*n* (%)]							
Not detained	131 (30.0)	40 (32.5)	17 (36.2)	1 (6.3)	33 (27.5)	24 (38.7)	*p* = 0.1439
Detained	306 (70.0)	85 (67.5)	30 (63.8)	15 (93.8)	87 (72.5)	38 (61.3)	
At risk [*n* (%)]							
No	15 (4.4)	4 (4.2)	3 (7.1)	0 (0)	3 (3.3)	3 (6.5)	*p* = 0.8321
Yes	328 (95.6)	91 (95.8)	39 (92.9)	12 (100)	89 (96.7)	43 (93.5)	
Diagnosis [*n* (%)]							
Psychopathic disorder	51 (12)	4 (3.2)	5 (11.1)	0 (0)	0 (0)	4 (6.6)	*p* < 0.001
Mental impairment	6 (1.4)	2 (2.4)	0 (0)	0 (0)	1 (0.9)	0 (0)	
Mental illness	319 (75.2)	111 (89.5)	37 (84.1)	16 (100)	109 (93.2)	55 (90.2)	
Multiple psychiatric diagnoses	21 (5.0)	0 (0)	0 (0)	0 (0)	2 (1.7)	0 (0)	
No confirmed diagnosis	27 (6.4)	7 (5.6)	2 (4.4)	0 (0)	5 (4.3)	2 (3.3)	

Age distribution of the sample was statistically significantly associated with ethnicity. There were more over-35s in the White ethnic group (66.7 %), Asian Indian (66 %) and African Carribean (60 %) groups, whilst more under 35s were in the Asian Pakistani (60.8 %), Asian Bangladeshi (87.5 %) and Black African (56.5 %) groups. Greater proportion of those living alone were in the African Caribbean (62.4 %) and White (50.4 %) groups, whilst all three Asian groups (Indian, Pakistani and Bangladeshi) had greater numbers living with others.

Proportion of community treatment order/recall (CTO/CTO recall) was highest amongst Asian Pakistani (7.9 %) and African Caribbean (9.2 %) groups, whilst police involvement and criminal justice referrals (Section 135/136) were highest amongst the Black African (16.1 %) group. Rates of psychopathic disorders (12 %), co-morbid disorders (5 %) and ‘no mental illness diagnosed’ (6.4 %) were highest in the White ethnic group. Within a diagnoses of ‘mental illness’, rates of schizophrenia-spectrum diagnosis were highest amongst Asian Bangladeshi, African Caribbean and Black African groups, and mood/affective disorders was highest amongst White (30.3 %), Asian Indian (25 %) and Asian Pakistani (24.2 %) groups accordingly.

### Proportion of assessments and detentions in the service user population

**Fig. 1:**
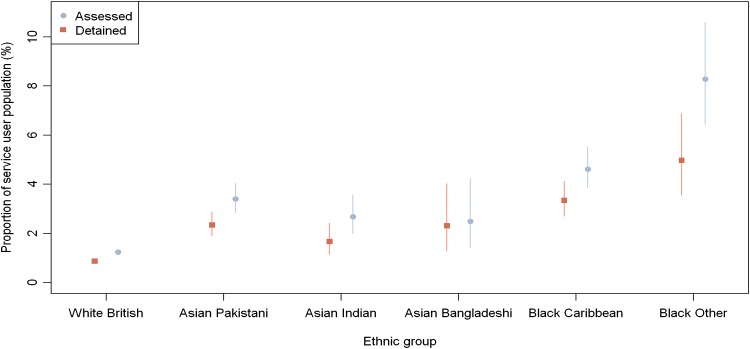
Proportion of assessments and detentions across six ethnic groups within the service user population

Figure 1 illustrates the proportion (%) of individuals assessed and detained within six ethnic groups in comparison with service users (*N* = 52,063) accessing the largest mental health trust in Birmingham from 2009 to 2010. The results reveal that a statistically greater proportion of service users from a BME background than those from the White ethnic group were assessed (*χ*^2^ = 416.22, *df* = 5, *p* < 0.001) and detained under the MHA (*χ*^2^ = 259.73, *df* = 5, *p* < 0.001). Post hoc analysis (Table 2) revealed that the proportion of individuals assessed and detained from all the BME groups within the service user population (apart from the Asian Bangladeshi group) was statistically greater than the proportion of White ethnic group. Within the BME groups, patients of Black African ethnicity were statistically more likely to get assessed and detained than those from any other ethnicity.

**Table 2:** Post-hoc analysis between proportion of assessments and detentions between different ethnic groups to the White British proportion

	Ethnic group	Proportion assessed (95 % confidence interval)	*χ* ^2^	*p* value	Proportion detained (95 % confidence interval)	*χ* ^2^	*p* value
Service-user population	White British/other	1.2 % (1.1, 1.4 %)	–	–	1.2 % (1.1, 1.4 %)	–	–
	Asian Pakistani	3.4 % (2.9, 4.0 %)	110	*	3.4 % (2.9, 4.0 %)	72.4	*
	Asian Indian	2.7 % (2.0, 3.6 %)	26.41	*	2.7 % (2.0, 3.6 %)	11.51	*
	Asian Bangladeshi	2.5 % (1.4, 4.2 %)	6.035	0.210	2.5 % (1.4, 4.2 %)	11.5	*
	African Caribbean	4.6 % (3.9, 5.5 %)	188.99	*	4.6 % (3.9, 5.5 %)	142.86	*
	Black African	8.3 % (6.4, 10.6 %)	254.2	*	8.3 % (6.4, 10.6 %)	122.82	*
Birmingham population	White British/Other	0.06 % (0.057, 0.069 %)	52.3	–	0.04 % (0.039, 0.049 %)	–	–
	Asian Pakistani	0.13 % (0.107, 0.153 %)	2.56	*	0.09 % (0.071, 0.109 %)	33.8	*
	Asian Indian	0.08 % (0.060, 0.108 %)	0.13	0.110	0.05 % (0.035, 0.073 %)	0.403	0.525
	Asian Bangladeshi	0.05 % (0.031, 0.095 %)	269.3	0.722	0.05 % (0.028, 0.090 %)	0.144	0.705
	African Caribbean	0.29 % (0.244, 0.351 %)	96.96	*	0.21 % (0.171, 0.263 %)	203.2	*
	Black African	0.23 % (0.174, 0.292 %)	52.3	*	0.14 % (0.096, 0.189 %)	43.50	*

* *p* < 0.001 (Bonferroni adjustment for 15 comparison tests)

### Proportion of assessments and detentions in Birmingham

**Fig. 2:**
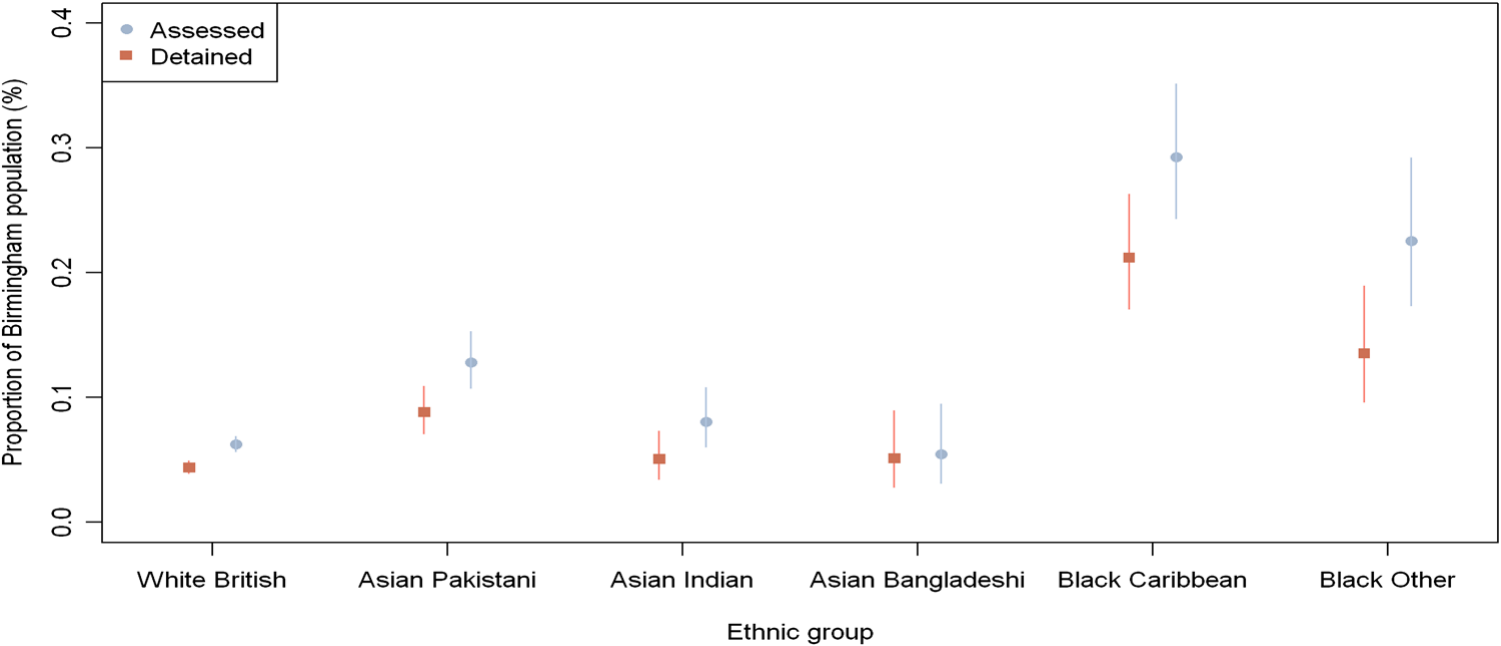
Proportion of assessments and detentions across six ethnic groups within Birmingham

Figure 2 illustrates the proportion (%) of individuals assessed and detained within six ethnic groups in comparison with the ethnic population estimates of Birmingham. There were statistically significant differences in the proportion of MHA assessments between ethnic groups in comparison with the ethnic population estimates of Birmingham in 2009 (*χ*^2^ = 336.78, *df* = 5, *p* < 0.001) and detentions in the ethnic population estimates of Birmingham in 2009 (*χ*^2^ = 232.30, *df* = 5, *p* < 0.001). Post hoc analysis (Table 2) revealed that the proportion of individuals assessed and detained from Asian Pakistani, African Caribbean, Black African ethnic groups within Birmingham were statistically greater than the proportion of White ethnic group.

### Ethnicity, multiple assessments and multiple detentions

Due to the non-normal distribution of data of multiple assessments and multiple detentions, Kruskal–Wallis one-way analysis of variance was used to test for overall difference. There were no ethnic differences within multiple assessments (*χ*^2^ = 3.815, *df* = 5, *p* = 0.576) and multiple detentions (*χ*^2^ = 5.248, *df* = 5, *p* = 0.386).

### Modelling the outcome of assessment

The results of the single regressions are shown in Table 3. The odds of the assessment resulting in a detention were statistically significantly increased if the service user was ‘at risk’ or over the age of 35. Diagnosis was also found to be statistically significantly associated with detention, with a ‘comorbid’ diagnoses reducing the odds of detention when compared to psychopathic disorders. However, a diagnosis of mental illness increased the odds.

**Table 3:** Single and multiple regression models of the outcome of a MHA assessment

Independent variable	Single regression models		Multiple regression model	
	OR	(95 % CI)	OR	(95 % CI)
Presence of risk				
Yes	87.989***	(12.039–643.061)	60.986***	(8.212–452.905)
Ethnicity				
White	1	Ref.	1	Ref.
Black	0.945	(0.688–1.297)	0.942	(0.626–1.416)
Asian	0.972	(0.714–1.324)	0.991	(0.659–1.492)
Other	0.650	(0.376–1.126)	0.505	(0.258–0.989)
Diagnosis				
Psychopathic disorder	1***	–	1***	
Mental impairment	0.774	(0.232–2.576)	1.359	(0.341–5.419)
Mental illness	1.614	(1.049–2.483)	1.599	(0.943–2.711)
Comorbid	0.774	(0.352–1.698)	0.907	(0.360–2.287)
None	0.224	(0.102–0.491)	0.334	(0.131–0.853)
Age				
Below 35	1*	–	1**	
35 and over	1.540	(1.203–1.971)	1.552	(1.130–2.131)
Living status				
Living alone	1	–	1*	
With others	0.807	(0.614–1.061)	0.732	(0.527–1.018)
NFA	1.038	(0.507–2.126)	1.252	(0.531–2.952)
Gender				
Male	1	–	1	
Female	0.057	(0.992–1.643)	1.192	(0.869–1.633)

Note that for variables with multiple categories, the significance noted on the reference category denotes the significance of the omnibus test for that variable

*OR* odds ratio, *CI* confidence interval, *Ref* reference

*** *p* < 0.001; **p < 0.01; * *p* < 0.05

In the multiple regression model of the total assessed population, the odds of detention were statistically significantly increased by having a mental illness, the presence of risk, being older than 35 years and living in supported accommodation. Most variables did not change behaviour from the single models with the exception of gender but neither model was statistically significant. Furthermore, ethnicity was also not found to alter the odds of detention under the MHA in either model with statistical significance (Table 3). We repeated the analysis, restricting the ethnicity breakdown to the three largest groups which also had the greatest disproportion in the rates of assessment and detention under MHA. These were Pakistani, Black Caribbean and Black African, compared with the White group. Ethnicity was still not associated with detention.

## Discussion

A greater proportion of BME groups, particularly African Caribbean and Black African were assessed and detained under the MHA (2007) than the White ethnic group. This was evident when the denominator was the regional general population or the population receiving care from secondary mental health services between April 2009 and March 2010. However when age, diagnosis, risk and level of social support were accounted for, ethnicity did not change the odds of MHA detention.

The ‘disproportionate’ excess of BME groups in the detained population could be explained by differences in rates of illness, presence of risk and level of social support. The BME excess in compulsory detentions has been attributed to several factors: some population related-higher rates of psychosis in the BME groups, lower or delayed help-seeking, mistrust of services and others service related factors such as misdiagnosis, ‘institutional racism’, poorer recognition at primary care level and hence a delayed, crisis presentation to services [8].

Our study found that both at the population level and mental health service use level, BME patients are more likely to be assessed and detained under MHA, and this excess was attributable to a diagnosis of mental illness, presence of risk and poorer level of social support. Although we still cannot definitely rule out the possibility that at every level, mental health services are ‘discriminatory’, our study adds to the accumulating evidence that the MHA excess was a function of higher rates of serious mental illnesses in the BME population. Recent studies have found that BME patients do not have a longer duration of untreated psychosis, hence there is no evidence of a delay in presentation to mental health care [20, 21]. The rate of criminal justice was greater amongst the Black African group in this study, which does indicate that more needs to be to improve mental health service engagement and assertive outreach to reduce the imposition of police involvement with minority ethnic groups; particularly the Black African and African Caribbean ethnic groups who are more likely to make contact with early intervention services through criminal justice involvement whereas White British patients access care through GP’s in the case of first episode psychosis [21].

This study also importantly revealed greater number of CTO/CTO recalls in Asian Pakistani and African Caribbean ethnic groups, which may be partly attributable to differential factors (age, diagnosis) in the two groups as suggested in our findings on ethnicity and the sample characteristics. Our findings are similar to those of Evans et al. [22], who reported an over representation of BME groups within the application of supervised CTOs, typically used with males around the age of forty and a primary diagnosis of psychosis. The differential rate of CTOs amongst ethnic groups raises serious clinical implications for service providers and users [22], as there is no evidence to support that compulsory supervision reduces the rate of readmission, particularly in patients with psychosis [23].

## Limitations

First, the dataset only looks at people who get to the assessment stage of the MHA. We have very limited information on what brought the person to this point. We have some limited information on their legal status at the time of the assessment, but with over half of the assessments occurred with the person “in the community”, it gives limited insight into factors preceding the assessment and if systematic bias was introduced at this pre-assessment stage, this analysis would not detect it. Hence, whilst our analyses do not show any evidence of ethnicity being a associated with detention at a MHA assessment, we cannot rule out the multiplicity of factors contributing to individuals being assessed, ethnicity being one of them.

Second, the proportion of assessments and detentions within a service user population assumes that every individual assessed and detained is accessing mental health services within Birmingham. It is unclear from our data, the true number of individuals that are service users. Also, the study does not have data on the number of assessments and detentions of individuals who are not permanently residing in Birmingham.

Third, information on MHA assessments conducted by various professionals (for example, Section 136), including approved clinicians prior to the involvement of an AMHP, but not subsequently completed under the MHA was beyond the scope of this study. Finally, interaction between ethnicity and probable moderating variables (for example, diagnosis) could not be computed in the multiple regression analysis due to the size of the standard error of the interaction effects.

Finally, Birmingham data may not explain population differences in other contexts, although a similar study that included data from London and Oxford also showed similar results [4]. Given the focus on ‘institutional racism’ in psychiatry and efforts to combat it [24], it is possible that clinical practices have changed over time and there is less discrimination within services. However we did not measure discriminatory attitudes or practices and cannot completely rule out such influences on the application of the MHA.

In conclusion, the study identifies sociodemodraphic (age, living status) and clinical factors (legal status at the time of assessment, diagnosis) that were statistically significantly associated with ethnicity amongst those assessed under the MHA 2007. Further research into factors that contribute to the increased risk of detention is required, integrating patient related information (e.g. help-seeking and pathways to care), their socio-cultural setting (e.g. socio-economic deprivation), clinical risk factors prior to admission (e.g. diagnosis) and service related factors (e.g. treatment provision and support) [8].

## References

[1] Dressing PDH, Salize HJ (2004). Compulsory admission of mentally ill patients in European Union Member States. Soc Psychiatry Psychiatr Epidemiol.

[2] Morgan C, Mallett R, Hutchinson G, Bagalkote H, Morgan K, Fearon P, Dazzan P, Boydell J, McKenzie K, Harrison G (2005). Pathways to care and ethnicity. 1: sample characteristics and compulsory admission report from the ÆSOP study. Br J Psychiatry.

[3] Salize HJ, Dressing H (2004). Epidemiology of involuntary placement of mentally ill people across the European Union. Br J Psychiatry.

[4] Singh SP, Burns T, Tyrer P, Islam Z, Parsons H, Crawford M (2014). Ethnicity as a predictor of detention under the Mental Health Act. Psychol Med.

[5] Vinkers D, De Vries S, Van Baars A, Mulder C (2010). Ethnicity and dangerousness criteria for court ordered admission to a psychiatric hospital. Soc Psychiatry Psychiatr Epidemiol.

[6] Keown P, Mercer G, Scott J (2008). Retrospective analysis of hospital episode statistics, involuntary admissions under the Mental Health Act 1983, and number of psychiatric beds in England 1996–2006. BMJ.

[7] Morgan C, Mallett R, Hutchinson G, Leff J (2004). Negative pathways to psychiatric care and ethnicity: the bridge between social science and psychiatry. Soc Sci Med.

[8] Singh SP, Greenwood N, White S, Churchill R (2007). Ethnicity and the mental health act 1983. Br J Psychiatry.

[9] Tolmac J, Hodes M (2004). Ethnic variation among adolescent psychiatric in-patients with psychotic disorders. Br J Psychiatry.

[10] Mann F, Fisher HL, Johnson S (2014). A systematic review of ethnic variations in hospital admission and compulsory detention in first-episode psychosis. J Ment Health.

[11] Bhui K, Stansfeld S, Hull S, Priebe S, Mole F, Feder G (2003). Ethnic variations in pathways to and use of specialist mental health services in the UK systematic review. Br J Psychiatry.

[12] Coid JW, Kahtan N, Gault S, Jarman B (2000). Ethnic differences in admissions to secure forensic psychiatry services. Br J Psychiatry.

[13] Leese M, Thornicroft G, Shaw J, Thomas S, Mohan R, Harty MA, Dolan M (2006). Ethnic differences among patients in high-security psychiatric hospitals in England. Br J Psychiatry.

[14] Lawlor C, Johnson S, Cole L, Howard LM (2012). Ethnic variations in pathways to acute care and compulsory detention for women experiencing a mental health crisis. Int J Soc Psychiatry.

[15] Corrigall R, Bhugra D (2013). The role of ethnicity and diagnosis in rates of adolescent psychiatric admission and compulsory detention: a longitudinal case-note study. J R Soc Med.

[16] Singh SP, Croudace T, Beck A, Harrison G (1997). Perceived ethnicity and the risk of compulsory admission. Soc Psychiatry Psychiatr Epidemiol.

[17] Raleigh VS, Irons R, Hawe E, Scobie S, Cook A, Reeves R, Petruckevitch A, Harrison J (2007). Ethnic variations in the experiences of mental health service users in England. Results of a national patient survey programme. Br J Psychiatry.

[18] Mann F, Fisher HL, Major B, Lawrence J, Tapfumaneyi A, Joyce J, Hinton MF, Johnson S (2014). Ethnic variations in compulsory detention and hospital admission for psychosis across four UK Early Intervention Services. BMC Psychiatry.

[19] Clarkson DB, Fan Y-A, Joe H (1993). A remark on algorithm 643: FEXACT: an algorithm for performing Fisher’s exact test in rxc contingency tables. ACM Trans Math Softw (TOMS).

[20] Morgan C, Fearon P, Hutchinson G, McKENZIE K, Lappin JM, Abdul-Al R, Morgan K, Dazzan P, Boydell J, Harrison G (2006). Duration of untreated psychosis and ethnicity in the AESOP first-onset psychosis study. Psychol Med.

[21] Ghali S, Fisher HL, Joyce J, Major B, Hobbs L, Soni S, Chisholm B, Rahaman N, Papada P, Lawrence J (2013). Ethnic variations in pathways into early intervention services for psychosis. Br J Psychiatry.

[22] Evans R, Makala J, Humphreys M, Mohan CR (2010). Supervised community treatment in Birmingham and Solihull: first 6 months. The Psychiatrist.

[23] Burns T, Rugkåsa J, Molodynski A, Dawson J, Yeeles K, Vazquez-Montes M, Voysey M, Sinclair J, Priebe S (2013). Community treatment orders for patients with psychosis (OCTET): a randomised controlled trial. Lancet.

[24] Singh SP, Burns T (2006). Race and mental health: there is more to race than racism. BMJ.

